# Veterans Health Administration Outpatient Psychiatry Staffing Model: Longitudinal Analysis on Mental Health Performance

**DOI:** 10.1007/s11606-023-08119-1

**Published:** 2023-06-20

**Authors:** Clifford Smith, Matthew Boden, Jodie Trafton

**Affiliations:** Veterans Health Administration, Washington, DC USA

**Keywords:** staffing model, staffing ratio, supply and demand

## Abstract

**Background:**

An adequate supply of mental health (MH) professionals is necessary to provide timely access to MH services. Veterans Health Administration (VHA) continues to prioritize the expansion of the MH workforce to meet increasing demand for services.

**Objective:**

Validated staffing models are essential to ensure timely access to care, to plan for future demand, to ensure delivery of high-quality care, and to balance the demands of fiscal responsibility and strategic priorities.

**Design:**

Longitudinal retrospective cohort of VHA outpatient psychiatry, fiscal years 2016–2021.

**Participants:**

Outpatient VHA psychiatrists.

**Main Measures:**

Quarterly outpatient staff-to-patient ratios (SPRs), defined as the number of full-time equivalent clinically assigned providers per 1000 veterans receiving outpatient MH care, were calculated. Longitudinal recursive partitioning models were created to identify optimal cut-offs for the outpatient psychiatry SPR associated with success on VHA’s measures of quality, access, and satisfaction.

**Key Results:**

Among outpatient psychiatry staff, the root node identified an outpatient SPR of 1.09 for overall performance (*p* < 0.001). For metrics associated with Population Coverage, a root node identified an SPR of 1.36 (*p* < 0.001). Metrics associated with continuity of care and satisfaction were associated with a root node of 1.10 and 1.07 (*p* < 0.001), respectively. In all analyses, the lowest SPRs were associated with the lowest group performance on VHA MH metrics of interest.

**Conclusions:**

Establishing validated staffing models associated with high-quality MH care is critical given the national psychiatry shortage and increasing demand for services. Analyses support VHA’s current recommended minimum outpatient psychiatry-specific SPR of 1.22 as a reasonable target to provide high-quality care, access, and satisfaction.

**Supplementary Information:**

The online version contains supplementary material available at 10.1007/s11606-023-08119-1.

Across America, the growing demand for mental health (MH) care far outpaces the current supply of MH providers.^1–3^ Specific to psychiatry, current provider deficits have been well documented and are projected to increase.^4–6^ The availability of an adequate workforce is critical to deliver safe and effective psychiatric care. With increasing demand and a limited supply of psychiatrists, it is vital to have clear staffing expectations to maintain efficient quality and access to care, while maintaining fiscal responsibility and stewardship.^7,8^

Within the Veterans Health Administration (VHA), between fiscal years (FY) 2006 and 2021, the number of outpatient MH visits increased from 10.7 to 20.0 million, and the proportion of veterans served by VHA who receive MH services increased from 20 to 28%. To meet the growing demand for MH services during this time, VHA increased overall MH staffing from 6923 to 18,606 full-time equivalents (FTE) and psychiatry-specific staffing from 1869 to 3439 FTE during this same period.

In an effort to align supply and demand with the optimal delivery of clinical care, numerous MH staffing models have been developed.^9–16^ While varied models have been proposed, no clinical or single industry standard identifies expected psychiatry staffing levels in private or public healthcare systems. In response to a 2012 US Department of Veterans Affairs Office of the Inspector General report,^17^ VHA established a recommended minimum outpatient MH staffing model focusing on the number of providers needed to treat a defined population. This recommendation (7.72 FTE per 1000 outpatient veterans) was calculated by averaging the overall outpatient staff-to-patient ratio (SPR) at that time. VHA subsequently established a psychiatry SPR of 1.22 FTE per 1000 veterans treated in outpatient MH by using the average outpatient psychiatry SPR at the time. To support these recommendations, VHA established internal dashboards to track SPRs at the facility level and formally demonstrated associations between outpatient SPR and MH performance.^18–20^

While greater MH staffing has been associated with decreased suicidal behavior among facility patients,^21–23^ the relationship between staffing levels and other outcomes, such as depression, has been mixed.^24,25^ In VHA, higher outpatient SPRs have been associated with better performance on VHA measures of quality, access, and satisfaction.^18–20^ Yet, with a national staff shortage, there are significant supply–demand challenges necessitating the need to optimize resources in a clinical and fiscally responsible manner. Ongoing research evaluating staffing models that optimally meet patient demand and provide positive MH outcomes given supply and fiscal limitations remains critical.

Optimal psychiatry SPRs that distinguish higher performing from lower performing VHA facilities have not been evaluated. To evaluate existing VHA psychiatry SPR recommendations, we conducted a series of exploratory analyses utilizing longitudinal binary recursive modeling to determine the optimal SPR associated with overall quality, access, and satisfaction. A series of decision tree models were developed using outpatient psychiatry SPR as the variable of interest. Optimal psychiatry SPR cut-offs have not been systematically evaluated, and this analytic approach can tell us whether the current VHA recommendation established in 2012 is consistent with statistically derived cut-offs for optimal quality, access, and satisfaction.

## METHODS

### Study Population and Data

This longitudinal study uses quarterly outpatient staffing data from FY 2016 through FY 2021. SPRs from each VHA Medical Center beginning the 1st quarter (Q1) of fiscal year (FY) 2016 (October, 2015) through the 4th quarter (Q4) of FY 2021 (September, 2021) were calculated.

### Measures

#### Staff-to-Patient Ratio

For each of the 24 quarters, outpatient psychiatry SPRs were calcuated.^18,19^ As calculated, psychiatry SPR does not reflect total FTE available at the medical center, but instead, the time psychiatrists spent in direct outpatient care; non-direct patient care time, such as program oversight, time spent in research or education, and time spent providing inpatient psychiatric services, is not included in the outpatient SPR.

#### MH Strategic Analytics for Improvement and Learning (MH SAIL) Domain Score

The Mental Health Strategic Analytics for Improvement and Learning (MH SAIL) Domain score is a meta-composite of three MH composite measures created by the VHA for internal evaluation.^26,27^ The three composite metrics consist of over 30 individual measures considered to assess (a) the proportion of veterans with identified mental disorders who receive MH services (termed Population Coverage), (b) the proportion of veterans receiving a treatment episode or coordinated, proactive care (termed Continuity of Care), and (c) ratings of veteran and provider opinions regarding access, quality, and coordination of care (termed Experience of Care). For each performance year (PY: FYQ4-FY + 1Q3), the individual metrics and the MH SAIL composite scores are standardized to the start of year facility distribution (mean = 0, in units of standard deviation) to assess individual facility change from baseline through the PY. For this study, analyses focused on MH SAIL Domain and the three composite measures, Population Coverage, Continuity of Care, and Experience of Care. See Table 1 in the Appendix for a description of the individual metrics comprising each MH SAIL composite for 2021 and score interpretation.

### Statistical Analyses

Traditional parametric analyses do not offer straightforward interpretation methods regarding staffing needed for optimal performance. Thus, we utilize unbiased random regression tree models to identify psychiatry SPRs associated with MH SAIL performance. Hypothesis testing through regression or factorial designs requires established groups or an a priori determination of group designation. In contrast, machine learning models, such as classification and regression trees (CART), offer computationally intensive, non-parametric statistical methods that have been shown to be advantageous when relationships are not necessarily linear.^28–30^ While CART-based algorithms function by maximizing a statistical criterion over all possible predictors and split points simultaneously, tree algorithm models developed by Horton, Hornik, and Zeileis^31^ correct the problem of selection bias and overfitting by choosing predictors for splitting based upon a series of tests identifying statistically significant associations between the responses and the predictors utilizing the conditional inference tree (cTree) framework.^30^ These methods^30^ have been extended to include mixed and random effect expectation maximization (REEM) models associated with longitudinal data.^32–34^ Fu and Simonoff^32^ subsequently combined the REEM tree algorithm with the conditional inference approach (REEMcTree) to recursively test the null hypothesis between random input and response variables by selecting the binary split based upon the highest *p*-value with the response variable.

To evaluate the relationships between outpatient psychiatry SPR and MH SAIL performance, we developed a series of unbiased conditional inference trees (REEMcTree). Use of the automated unbiased conditional inference tree modeling analytic approach will tell us which SPR cut-offs are associated with statistically significant group differences in MH SAIL performance. All REEMcTree models are fitted using a maximum likelihood, random effect intercept model allowing for variations across facilities. Analyses were completed in R 4.2.1^35^ utilizing psych, party, and ggplot2 packages.^36–39^ REEMcTree coding is available at http://people.stern.nyu.edu/jsimonof/unbiasedREEM/.

## RESULTS

### Psychiatry Staffing

**Figure 1:**
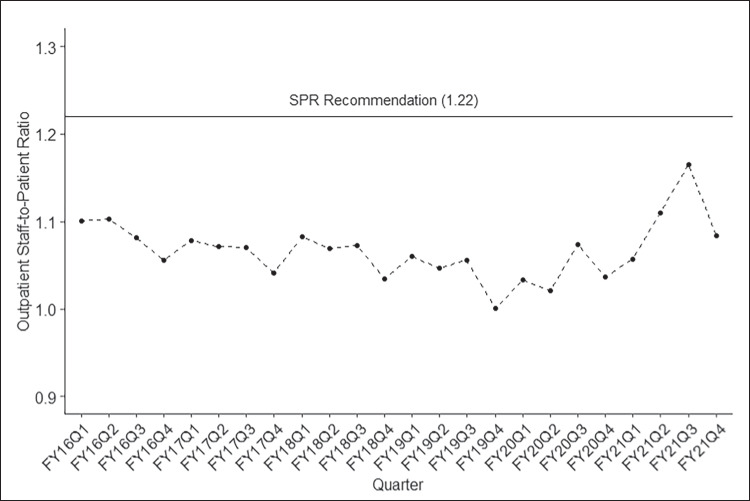
Longitudinal trend outpatient psychiatry staff-to-patient ratio.

For the 24 quarters between FY16Q1 and FY2021Q4, the number of onboard psychiatrists increased from 3209 to 3439 staff. Despite ongoing hiring psychiatry across the 6 years, SPR remained generally flat across time (Fig. 1), as the number of unique veterans receiving MH services increased from 1.63 million to nearly 1.84 million over this period. Quarterly SPRs and MH SAIL performance are available in Table 1 in the Appendix.

### Psychiatry Staffing Ratio

**Figure 2:**
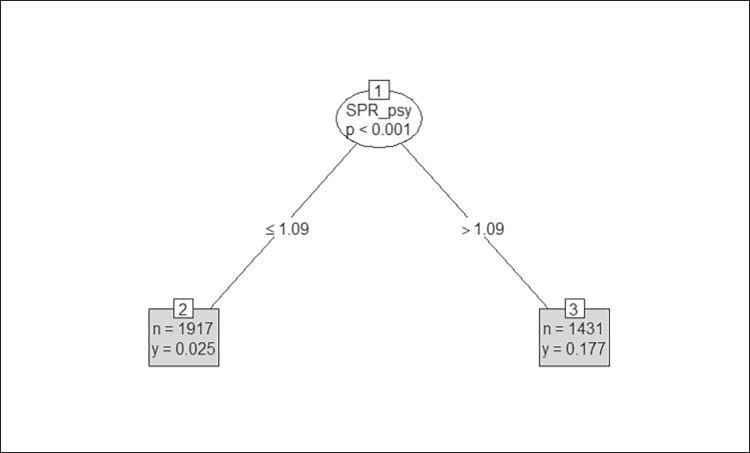
Optimal psychiatry staff-to-patient ratio for MH SAIL Domain performance. Note. Longitudinal REEM conditional inference tree for psychiatry staff-to-patient ratio with MH overall SAIL performance. Each terminal node shows two figures, the first (*n*) stating the number of observations falling in the branch and the second (*y*) giving the mean value for MH SAIL Domain in the branch.

The recursive model for overall MH SAIL Domain performance identified an outpatient psychiatry SPR of 1.09 as the initial split (Fig. 2). The average MH SAIL Domain performance was.02 for sites below the 1.09 average, and 0.18 for sites above the average. The initial root node for optimal performance on the Population Coverage domain (see Fig. 1 in the Appendix) was an outpatient psychiatry SPR of 1.36 with lower SPRs associated with decreased performance (leaf node 2: mean =  − 0.06) compared to sites with psychiatry SPRs above this level (leaf node 3: mean = 0.08). Unbiased conditional decision tree for Continuity of Care identified a single psychiatry-specific SPR associated with group performance differences of 1.10 (see Fig. 2 in the Appendix). Facilities below this level averaged 0.11 on MH SAIL Continuity of Care while facilities above this level averaged 0.27. The decision tree model identified a single SPR split at 1.07 FTE per 1000 outpatient MH patients with sites averaging Experience of Care performance of − 0.02 below this SPR and sites averaging 0.14 above this SPR (see Fig. 3 in the Appendix).

## CONCLUSION

Numerous MH staffing models have been developed^9–16^ to balance provider supply with clinical demand. Recognizing the critical need for MH staff and the clear relationship between supply and demand, congressional legislation has demonstrated ongoing interest in the VHA staffing model and the ratio of MH providers to veterans receiving care in the VHA. In response to a 2012 OIG report, VHA recommended a minimum overall outpatient MH staffing ratio of 7.72 and an outpatient psychiatry SPR of 1.22. In the current study, we identified optimal outpatient psychiatry SPRs associated with VHA measures of MH quality, access, and satisfaction utilizing non-parametric binary recursive models. Overall, the current study found that higher outpatient psychiatry SPRs are associated with higher MH SAIL performance. Optimal SPRs varied for the individual MH SAIL composites, which is not unexpected. In MH SAIL, Population Coverage is specifically designed to assess overall access to care utilizing a population health approach (i.e., engagement of known population in care), whereas the Continuity of Care domain assesses the ongoing ability of those engaged in care to receive care.^26,27^ Given the complementary, but unique demands of care within each domain, the staffing levels needed to meet those demands are unique to each domain. Regardless, results were consistent across MH SAIL domains, supporting 1.22 as a reasonable balance between the models and the VHA recommendation as minimum psychiatry SPR benchmark.

The principle of supply and demand is commonly applied to healthcare access. In VHA, efforts to increase supply (i.e., hiring additional providers, increasing daily productivity expectations, increasing scheduling utilization efficiency, etc.) have been offset, paradoxically, by further demand increases (i.e., more veterans requesting care). This paradox is not unexpected for two reasons. First, the lack of growth in psychiatry SPR despite increased VHA hiring is consistent with the MH services dilemma outlined by Riessman^40^ who observed that if the supply of a MH service is increased to meet the growing demand, this will in turn lead to even further increase in demand. This paradox has been the experience across the VHA, particularly at facilities with lower SPR levels. This increased demand is thought to reflect the insufficient supply–demand balance now having additional resources. Second, while VHA has implemented initiatives to increase provider efficiency, such as scheduling procedures and bookable time expectations, Jevons’ Paradox^41^ asserts that increasing the efficiency with which a resource is available tends to increase, rather than decrease, the rate of consumption of that resource. While originally conceptualized in relation to material resource utilization, this observation is consistent with recent VHA experiences in which access initiatives have resulted in minimal access gains due to the offset of increased patient demand for and utilization of those services. Similar to the observation by Riessman,^40^ increased clinic efficiency addressing insufficient supply–demand balance when optimized has resulted in minimal access gains, as the unmet, historical demand quickly utilizes available resources. Taking these observations into account, the pace of hiring or supply enhancement must aggressively stay ahead of the corresponding increases in patient care demand.

This exploratory analysis utilizing binary recursive modeling is not without limitation. First, performance on MH SAIL is variable across the SPR spectrum. There are low performing facilities with high SPR levels and high performing facilities with low SPR levels, highlighting that staffing alone is not sufficient for MH SAIL success. Factors such as leadership and administrative decisions, including program alignment, team-based collaboration, and provider burnout, among others, likely contribute to MH SAIL variety. Second, MH SAIL is specifically designed to be fluid in that new measures are added, existing measures are updated, and stable measures associated with program success are removed upon annual review. While the decision tree modeling process independently considers individual SPR and MH SAIL, optimal SPR for any given year may vary slightly as measures of quality, access, and satisfaction are added or removed. Third, moderating covariates were not utilized in the decision tree models, as we focused on the overall psychiatry SPR recommendation. Covariates, such as hospital complexity, staff-mix^42^, case-mix, and rurality, likely impact supply–demand balance and MH SAIL performance. Currently, while VHA policy does not recommend a minimum staffing ratio based upon potential covariates, additional research will be necessary to refine optimal resource alignment. Finally, this longitudinal analysis consists of staffing and performance data collected across pre- and post-COVID pandemic periods, which is another potential covariate moderating SPRs. VHA shifted rapidly to telehealth services in an effort to maintain safety for veterans and staff,^43^ and VHA MH SAIL metrics were updated to include the expanded telehealth delivery services authorized by the Centers for Medicare and Medicaid Services (CMS). As with additional covariates, there may be internal and external contributors to optimal performance during any given performance year, which requires additional research consideration.

Broadly, there are two ways to increase the outpatient SPR. First, focusing on the numerator, you can increase SPR by adding more staff either through hiring or by administrative decisions, such as shifting psychiatrists from non-outpatient settings (e.g., acute inpatient units or residential units) or by decreasing psychiatrist administrative, research, or teaching availability. Second, focusing on the denominator, outpatient SPRs can be increased by reducing the demand or number of patients who utilize MH care. Factors limiting utilization may be attributed to internal (e.g., eliminating services, increasing barriers to engagement, or restricting eligibility) or external (e.g., reducing patient treatment-seeking patterns associated with a national pandemic) causes. VHA wants to enhance (i.e., increase) utilization and access across the spectrum of care; thus, increasing the SPR numerator by shifting staff resources or decreasing the denominator by reducing the volume of patients does not align with VHA goals.

Supply and demand models designed to optimize care must evaluate opportunities to optimize the supply and address the demand. To increase the supply of providers, the first strategy typically focuses on hiring. In 2017, VHA established the Mental Health Hiring Initiative (MHHI) to hire 1000 net new MH providers across the enterprise. The MHHI has since developed into a Sustainment Initiative focused on enhancing hiring efficiencies across the enterprise. Between 2016 and 2021, VHA increased psychiatry FTE by 187 FTE (5.75%). Hiring efforts to date have included establishing dedicated recruiters, increasing advertising of positions, increasing trainee and fellowship opportunities, increasing the number of available debt reduction programs, increasing salary pay scales, and leveraging telehealth and alternate work schedules. Recent congressional legislation requiring VHA MH staffing models and annual reports on underserved and critical hiring sites, use of alternative work schedules, and increased trainee/resident funding will continue to prioritize MH staffing levels and the need for ongoing hiring across the VHA. Additional strategies in VHA have included expanding MH support services through hiring Peer Support Specialists, refining processes for referral to ensure the right care at the right time is available, utilizing stepped care and care management practices, and maximizing services supported by advanced practice providers, including clinical pharmacists and nurse practitioners, among others.

Strategies for managing demand typically focus on the volume of patients requesting care. Recent congressional legislation has increased veteran and caregiver eligibility for MH services. Demand management strategies, such as making a program difficult to engage, limiting hours of availability, and restricting eligibility, can reduce service demand, yet these efforts are clinically inappropriate. In contrast, there are clinically appropriate strategies for managing demand. For example, VHA developed medical decision algorithms to identify veterans receiving psychiatric management who are medically stable and ready for discharge back to lower level of care for ongoing medication management. Clinical decision tools are available to prioritize outreach to at-risk veterans, which may subsequently reduce increased service needs due to a crisis. Additionally, VHA has moved to train MH staff in time-limited, evidence-based psychotherapy protocols and has emphasized the delivery of an episode of care where appropriate. While many veterans may require intensive, life-long psychiatric management, many veterans may have their MH needs met through a variety of strategies, such as Peer Support Services, problem focused, brief intervention in Primary Care Mental Health Integration, utilization of group practices, including drop-in group medical appointments, and increasing asynchronous care delivery. Hiring is certainly a strategy for increasing the supply of available providers, yet hiring alone will not solve the supply–demand imbalance. Strategies to both increase provider availability and manage growing demand in a clinically efficient manner will be critical for maximizing veteran MH care.

The outpatient psychiatry SPR utilized in the VHA could certainly be applied to non-VHA settings. While the VHA healthcare system includes a broad array of integrated physical, social, and MH resources not common to private healthcare systems, the core principle of calculating the necessary resources (supply) needed to meet a known clinical need (demand) is universal—the provision of MH care is dependent upon staff availability to meet the patient demand. VHA methodology integrates caseload calculations,^9^ needs-based calculations,^13,14^ and ratio consieration.^12,16^ In contrast to previous studies where ratios are reported as full-time equivalent (FTE) providers per 100,000 health organization members,^12,16^ the VHA SPR focuses on the number of providers directly needed to treat MH outpatients (per 1000), which is more conducive to facility-level planning and supports efforts to ensure adequacy of MH services for patients who engage. Additional provider time necessary for program administration and non-direct clinical duties, such as research and education training, is over and above the direct care SPR. While non-VHA systems may not have the volume, care complexity, or fiscal and logistical resources of the VHA, all systems must work to optimize supply limitations and demand expectations. Future research utilizing non-VHA healthcare systems would need to confirm or refute this assertion.

## Supplementary Information

Below is the link to the electronic supplementary material.

Supplementary file1 (DOCX 31 kb)

Supplementary file2 (DOCX 30 kb)

Supplementary file3 (DOCX 31 kb)

Supplementary file4 (DOCX 18 kb)

Supplementary file5 (DOCX 42 kb)
