# Supplementary material for: Veterans Health Administration Outpatient Psychiatry Staffing Model: Longitudinal Analysis on Mental Health Performance
Source: J Gen Intern Med. 2023 Jun 20;38(Suppl 3):814–20. doi: 10.1007/s11606-023-08119-1 (PMC10356727; doi:10.1007/s11606-023-08119-1)
Supplement: Supplementary file 1 — Supplementary file1 (DOCX 31 kb) [file 11606_2023_8119_MOESM1_ESM.docx]

**Figure 1**

*Optimal Psychiatry Staff-to-Patient Ratio for MH SAIL Population Coverage*


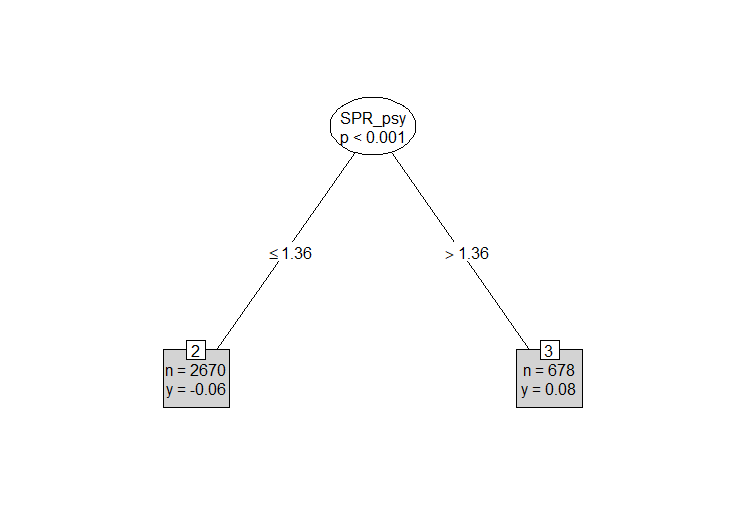


Note. Longitudinal REEM conditional inference tree for staff-to-patient ratio associated with MH SAIL Population Coverage performance. Each box in the terminal nodes shows two figures, the first (n) stating the number of observations falling in the branch and the second (y) giving the mean value of MH SAIL Population Coverage in the branch.
