# Supplementary material for: Veterans Health Administration Outpatient Psychiatry Staffing Model: Longitudinal Analysis on Mental Health Performance
Source: J Gen Intern Med. 2023 Jun 20;38(Suppl 3):814–20. doi: 10.1007/s11606-023-08119-1 (PMC10356727; doi:10.1007/s11606-023-08119-1)
Supplement: Supplementary file 3 — Supplementary file3 (DOCX 31 kb) [file 11606_2023_8119_MOESM3_ESM.docx]

**Figure 3**

*Optimal Staff-to-Patient Ratio for MH SAIL Experience of Care*


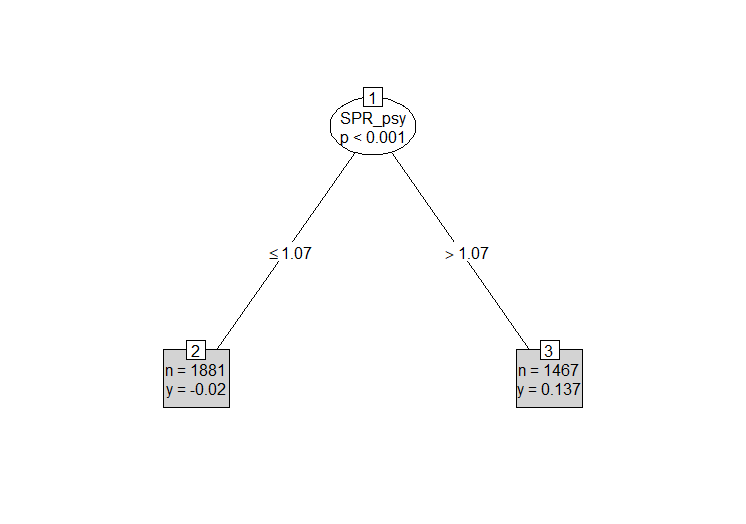


Note. Longitudinal REEM conditional inference tree for staff-to-patient ratio associated with MH SAIL Experience of Care performance. Each box in the terminal nodes shows two figures, the first (n) stating the number of observations falling in the branch and the second (y) giving the mean value of MH SAIL Experience of Care in the branch.
