# Supplementary material for: Veterans Health Administration Outpatient Psychiatry Staffing Model: Longitudinal Analysis on Mental Health Performance
Source: J Gen Intern Med. 2023 Jun 20;38(Suppl 3):814–20. doi: 10.1007/s11606-023-08119-1 (PMC10356727; doi:10.1007/s11606-023-08119-1)
Supplement: Supplementary file 4 — Supplementary file4 (DOCX 18 kb) [file 11606_2023_8119_MOESM4_ESM.docx]

Table 1

2021 MH SAIL Composite Measures

| **Population Coverage Composite** | **Continuity of Care Composite** | **Experience of Care** |
| --- | --- | --- |
|  |  |  |
| Percent of MH-treated patients with a family psychotherapy visit | Percent of veterans with ICMHR-targeted diagnosis and services with at least 12 ICMHR visits in the past 90 days | MH Provider Survey--Timely Access MH Care scale |
| Percent of veterans with ICMHR-targeted diagnosis receiving ICMHR services | Percent of veterans with PRRC-targeted diagnosis and services with at least 3 PRRC visits in the past 90 days | MH Provider Survey--Collaborative MH Care scale |
| Percent of veterans with PRRC-targeted diagnosis served by PRRC | Care process composite for Veterans at high risk for suicide | MH Provider Survey--Quality of MH Care scale |
| Percent VA enrollees using alcohol and other drug services | Percent of veterans with SMI diagnosis who have an assigned primary care provider and a primary care visit | MH Provider Survey--Job Satisfaction scale |
| Percent of veterans using MH services | Percent high-risk veterans with SMI diagnosis who have a MH visit every 6 months | Veteran Satisfaction Survey--MH Appointment Access scale |
| Percent Primary Care Patients engaged in Primary Care-Mental Health Integration | Percent Inpatient and residential MH discharges with outpatient MH care engagement within 30 days post-discharge | Veteran Satisfaction Survey--Patient-Centered MH Care scale |
| Percent of veterans with Primary Care-Mental Health Integration, Same Day Access for Initial Care | Percent of veterans with depression-diagnosis and treated veterans with 5 psychotherapy visits in 10 weeks |  |
| Percent of veterans with MH diagnosis who have a MH E&M encounter | Percent of veterans with SMI-diagnosis and treated veterans with 5 psychosocial treatment visits in 10 weeks |  |
| Percent MH-service-connected veterans in the facility catchment area with MH care | Percent of veterans with SUD-diagnosis and treated veterans with 4 psychosocial treatment visits in 8 weeks |  |
| Percent of veterans with depression diagnosis with psychotherapy visit for depression | Percent of veterans with PTSD-diagnosis and treated veterans with 5 psychotherapy visits in 10 weeks |  |
| Percent of veterans with SMI diagnosis with psychosocial treatment for SMI | Percent of veterans diagnosed with schizophrenia, bipolar disorder, or other psychoses using supported employment services with 3 SE visits in the last 90 days |  |
| Percent of veterans with SUD diagnosis with psychosocial treatment for SUD |  |  |
| Percent of veterans with PTSD diagnosis with psychotherapy visit for PTSD |  |  |
| Percent of veterans with opioid use disorder diagnosis who received M-OUD |  |  |
| Percent of veterans with SUD diagnosis with intensive SUD treatment |  |  |

Note. MH = Mental Health, SAIL = Strategic Analytics for Improvement and Learning, ICMHR = Intensive Community Mental Health Recovery, PRRC = Psychosocial Rehabilitation and Recovery Center, SUD = Substance Use Disorder, SMI = Serious Mental Illness, M-OUD = Medications for opioid use disorder, PTSD = Post Traumatic Stress Disorder, SE = Supported Employment, E&M = Evaluation and Management

Scoring: Each measure within a composite is scored per the measure description. In the first quarter of the performance year (i.e. quarter 4 of the fiscal year), the facility mean and standard deviation for each measure is calculated. Within that performance year (i.e. fiscal year quarter 4 through the following fiscal year quarter 3), individual measure scores are standardized as: (“Measure score” minus “quarter 4 facility mean”) divided by “quarter 4 facility standard deviation”. This provides standardized scores as a difference from baseline quarter facility mean in units of baseline standard deviation. These standardized scores allow for both simplified review of relative performance across facilities, and improvement from baseline over the performance year. Composite scores for Population Coverage, Continuity of Care and Experience of Care are calculated as a weighted average of standardized scores across all measures in the composite. The MH Domain score is an average of these 3 composite scores.
