# Supplementary material for: Veterans Health Administration Outpatient Psychiatry Staffing Model: Longitudinal Analysis on Mental Health Performance
Source: J Gen Intern Med. 2023 Jun 20;38(Suppl 3):814–20. doi: 10.1007/s11606-023-08119-1 (PMC10356727; doi:10.1007/s11606-023-08119-1)
Supplement: Supplementary file 5 — Supplementary file5 (DOCX 42 kb) [file 11606_2023_8119_MOESM5_ESM.docx]

Table 1

Descriptive Statistics by Quarter

Variable Time Mean (SD) Min Max Median

SPR_psy FY16Q1 1.10 (0.37) 0.33 2.17 1.07

FY16Q2 1.10 (0.36) 0.0 2.00 1.10

FY16Q3 1.08 (0.36) 0.44 1.98 1.04

FY16Q4 1.06 (0.36) 0.36 2.09 1.02

FY17Q1 1.08 (0.37) 0.19 2.07 1.04

FY17Q2 1.07 (0.36) 0.09 2.07 1.03

FY17Q3 1.07 (0.35) 0.15 2.02 1.00

FY17Q4 1.04 (0.35) 0.29 2.05 0.97

FY18Q1 1.08 (0.36) 0.21 2.19 1.04

FY18Q2 1.07 (0.35) 0.20 2.14 1.00

FY18Q3 1.08 (0.35) 0.20 2.13 1.03

FY18Q4 1.03 (0.34) 0.20 1.93 1.00

FY19Q1 1.06 (0.37) 0.17 2.03 1.00

FY19Q2 1.05 (0.35) 0.18 2.06 0.97

FY19Q3 1.06 (0.32) 0.32 2.04 1.02

FY19Q4 1.00 (0.31) 0.38 1.87 0.97

FY20Q1 1.03 (0.34) 0.29 2.19 0.99

FY20Q2 1.02 (0.36) 0.37 2.59 0.96

FY20Q3 1.07 (0.40) 0.37 2.30 1.01

FY20Q4 1.04 (0.37) 0.38 2.12 0.97

FY21Q1 1.06 (0.40) 0.32 2.11 0.99

FY21Q2 1.11 (0.40) 0.32 2.25 1.03

FY21Q3 1.17 (0.43) 0.19 2.54 1.09

FY21Q4 1.08 (0.39) 0.23 2.28 1.05

SAIL_MHDom FY16Q1 0.17 (1.00) -2.30 3.45 0.12

FY16Q2 0.24 (0.98) -2.47 3.15 0.23

FY16Q3 0.34 (0.93) -1.81 2.84 0.32

FY16Q4 0.00 (1.00) -2.34 3.03 -0.18

FY17Q1 0.10 (0.99) -2.39 3.17 0.01

FY17Q2 0.12 (1.01) -2.48 3.48 0.08

FY17Q3 0.19 (0.98) -2.31 3.32 0.17

FY17Q4 0.00 (1.00) -2.18 2.48 -0.09

FY18Q1 0.16 (0.99) -2.04 2.70 0.07

FY18Q2 0.18 (0.98) -2.18 2.46 0.01

FY18Q3 0.40 (0.96) -2.21 2.68 0.33

FY18Q4 0.00 (1.00) -2.15 2.37 -0.18

FY19Q1 0.18 (0.98) -2.14 2.87 -0.01

FY19Q2 0.26 (1.00) -2.32 3.10 0.13

FY19Q3 0.30 (1.01) -2.57 3.64 0.14

FY19Q4 -0.02 (0.98) -2.97 3.18 -0.13

FY20Q1 -0.06 (1.00) -2.80 2.90 -0.17

FY20Q2 -0.07 (0.98) -2.28 2.40 -0.23

FY20Q3 -0.49 (0.96) -2.71 1.94 -0.59

FY20Q4 0.00 (1.00) -2.24 2.29 -0.56

FY21Q1 -0.06 (1.00) -2.84 2.86 -0.17

FY21Q2 -0.10 (1.04) -3.00 2.43 1.04

FY21Q3 0.25 (1.12) -2.57 2.97 0.09

FY21Q4 0.00 (0.73) -1.40 1.77 0.73

SAIL_PopCov FY16Q1 0.17 (0.98) -1.85 3.33 0.13

FY16Q2 0.27 (0.97) -1.68 3.31 0.23

FY16Q3 0.35 (0.97) -1.62 3.21 0.27

FY16Q4 0.00 (1.00) -2.09 2.69 -0.08

FY17Q1 -0.01 (0.99) -2.24 2.55 -0.11

FY17Q2 -0.02 (0.98) -2.30 2.50 -0.12

FY17Q3 -0.04 (0.96) -2.32 2.44 -0.17

FY17Q4 0.00 (1.00) -2.36 2.52 -0.12

FY18Q1 -0.02 (1.00) -2.35 2.69 -0.14

FY18Q2 -0.05 (0.97) -2.34 2.73 -0.19

FY18Q3 -0.05 (0.97) -2.65 2.83 -0.19

FY18Q4 0.00 (1.00) -2.43 2.58 -0.15

FY19Q1 0.06 (0.98) -2.35 2.75 -0.07

FY19Q2 0.08 (0.98) -2.42 2.88 -0.06

FY19Q3 0.07 (0.99) -2.52 2.84 -0.07

FY19Q4 -0.01 (0.99) -2.60 2.66 -0.11

FY20Q1 -0.04 (1.01) -2.54 2.83 -0.10

FY20Q2 -0.10 (0.99) -2.66 2.92 -0.17

FY20Q3 -0.48 (0.97) -2.71 2.53 -0.64

FY20Q4 0.00 (1.00) -2.55 2.97 -0.12

FY21Q1 -0.04 (1.01) -2.54 2.83 -0.10

FY21Q2 -0.63 (0.99) -2.66 2.16 -0.74

FY21Q3 -0.31 (1.00) -2.38 2.67 -0.41

FY21Q4 -0.01 (0.44) -1.04 1.37 -0.05

SAIL_ContCare FY16Q1 0.04 (1.06) -2.30 2.82 0.04

FY16Q2 0.09 (1.06) -2.52 2.69 0.21

FY16Q3 0.13 (0.96) -1.88 2.64 0.19

FY16Q4 0.00 (1.00) -2.38 2.70 -0.09

FY17Q1 0.20 (1.01) -2.16 2.84 0.17

FY17Q2 0.27 (1.04) -3.05 2.94 0.30

FY17Q3 0.30 (1.06) -4.31 2.92 0.42

FY17Q4 0.00 (1.00) -2.46 2.42 0.10

FY18Q1 0.38 (0.98) -1.88 2.84 0.40

FY18Q2 0.47 (0.98) -1.94 2.85 0.43

FY18Q3 0.94 (0.99) -2.09 3.34 0.92

FY18Q4 0.00 (1.00) -2.11 2.70 -0.06

FY19Q1 0.30 (1.00) -2.14 3.15 0.27

FY19Q2 0.43 (1.04) -2.19 3.39 0.46

FY19Q3 0.57 (1.01) -2.46 4.25 0.60

FY19Q4 -0.01 (0.99) -2.72 3.62 -0.10

FY20Q1 -0.03 (0.98) -2.78 3.01 0.00

FY20Q2 -0.04 (0.94) -2.08 2.66 0.94

FY20Q3 -0.65 (1.03) -2.77 2.74 -0.76

FY20Q4 0.00 (1.00) -2.47 2.69 0.05

FY21Q1 -0.03 (0.98) -2.78 3.01 0.00

FY21Q2 0.33 (1.11) -3.00 3.00 1.02

FY21Q3 0.48 (1.06) -3.00 3.00 0.52

FY21Q4 -0.07 (0.43) -1.42 0.97 -0.07

SAIL_ExpCare FY16Q1 0.15 (0.95) -2.62 3.49 0.14

FY16Q2 0.15 (0.95) -2.62 3.49 0.14

FY16Q3 0.25 (0.94) -2.46 3.30 0.32

FY16Q4 0.00 (1.00) -2.90 3.27 0.06

FY17Q1 0.02 (1.00) -3.32 3.05 0.05

FY17Q2 0.02 (1.00) -3.32 3.05 0.05

FY17Q3 0.14 (0.99) -2.48 2.94 0.19

FY17Q4 0.00 (1.00) -2.67 2.82 0.03

FY18Q1 -0.01 (0.96) -2.30 3.04 -0.01

FY18Q2 -0.01 (0.96) -2.30 3.04 -0.01

FY18Q3 -0.01 (0.95) -2.70 3.02 -0.09

FY18Q4 0.00 (1.00) -2.70 3.02 -0.12

FY19Q1 0.03 (1.00) -2.30 2.90 -0.10

FY19Q2 0.04 (1.00) -1.99 2.47 0.03

FY19Q3 0.00 (0.98) -2.29 2.60 -0.02

FY19Q4 -0.01 (1.00) -2.51 2.17 -0.07

FY20Q1 -0.07 (0.98) -2.46 2.23 -0.15

FY20Q2 -0.02 (0.97) -3.26 2.27 0.06

FY20Q3 0.07 (0.88) -2.34 2.08 0.10

FY20Q4 0.00 (1.00) -2.54 2.56 -0.10

FY21Q1 -0.07 (0.98) -2.46 2.23 0.05

FY21Q2 0.08 (1.08) -2.72 2.96 1.02

FY21Q3 0.35 (1.21) -2.90 3.00 0.23

FY21Q4 0.00 (0.75) -1.87 2.14 0.00

Note. FY = Fiscal Year; Q = Quarter; SPR_psy = Psychiatry Staff-to-Patient-Ratio.
